# Building deep learning models for evidence classification from the open access biomedical literature

**DOI:** 10.1093/database/baz034

**Published:** 2019-04-02

**Authors:** Gully A Burns, Xiangci Li, Nanyun Peng

**Affiliations:** 1 Chan Zuckerberg Initiative, Redwood City, CA, USA; 2 Information Sciences Institute, Viterbi School of Engineering, University of Southern California, Marina del Rey, CA, USA

## Abstract

We investigate the application of deep learning to biocuration tasks that involve classification of text associated with biomedical evidence in primary research articles. We developed a large-scale corpus of molecular papers derived from PubMed and PubMed Central open access records and used it to train deep learning word embeddings under the GloVe, FastText and ELMo algorithms. We applied those models to a distant supervised method classification task based on text from figure captions or fragments surrounding references to figures in the main text using a variety or models and parameterizations. We then developed document classification (triage) methods for molecular interaction papers by using deep learning mechanisms of attention to aggregate classification-based decisions over selected paragraphs in the document. We were able to obtain triage performance with an accuracy of 0.82 using a combined convolutional neural network, bi-directional long short-term memory architecture augmented by attention to produce a single decision for triage. In this work, we hope to encourage biocuration systems developers to apply deep learning methods to their specialized tasks by repurposing large-scale word embedding to apply to their data.

## Introduction

Modern deep learning systems provide powerful machine learning capabilities that do not require extensive feature engineering to provide state-of-the-art performance (1). Although deep learning methods derive their power from the use of very large quantities of training data required to build models, their utility stems from their ability to learn general-purpose representations from large unlabeled corpora (2). These representations provide structured input for subsequent machine learning analysis that effectively captures lexical semantics without requiring researchers to use natural language processing (NLP) approaches to represent that meaning explicitly as features. The popularization of industrial-standard open toolkits for deep learning further supports researchers and developers in developing machine learning components for biocuration tasks. We here describe deep learning methods focused on the classification of scientific text directly describing experimental evidence for some tasks relevant to biocuration (identifying experimental methods and article triage). We focus on biological work studying molecular interaction.

The first impactful work on developing word embedding representation for large-scale biomedical corpora was performed by Pysallo *et al.* where they trained so-called Word2Vec models (2) on a collection of large-scale biomedical technical corpora made of the entire PubMed database, the open access collection from PubMed Central (PMC) and Wikipedia (3). This paper has a disproportionately large impact (in terms of citations) due largely to the fact that the authors shared the representations online as open access data, enabling other researchers to build effective neural network systems for a range of tasks [see (4–6) for examples]. A variety of different computational methods for generating word representations have subsequently been developed. The GloVe algorithm attempts to learn word vectors so that their distance reflects words’ semantic similarities (7). Facebook’s FastText algorithm uses subword information to augment performance for previously unseen words (8). Deep contextualized word representations (‘ELMo’) from AI2 provide a deeper representation that encodes the context information and outperforms other methods at several domain-independent benchmark tasks (9). Very recently, the ‘Bidirectional Encoder Representations from Transformers’ (BERT) architecture has built upon and surpassed even these prior results across a range of tasks (10). We have not yet had the opportunity to build and apply BERT models to our data.

In this paper, we seek to investigate the use of deep learning on specialized biocuration-specific tasks, taking into account (i) how these tasks differ from standard information retrieval (IR) and information extraction (IE) tasks, (ii) the specialized nature of biomedical scientific language and (iii) the comparatively limited resources of academic bioinformatics research teams in developing deep learning models for biocuration tasks.

Past evaluations of the application of deep learning to biomedical tasks use standard NLP tasks such as IR and IE to describe improvements made by deep learning methods (11). We specifically describe tasks designed to develop text-mining tools to enable biocuration. These tools typically center around supporting human-centered tasks that are time consuming to execute in a typical workflow (12). ‘Document triage’, the task of identifying which papers should be prioritized for detailed examination, is a key step that we have previously investigated with non-neural methods (13, 14). Recent work has demonstrated deep learning can accelerate triage in genome-wide association studies based on input data from PubMed and Pubtator services (15). Other important work in this area focuses on using deep learning to provide matching functions that function at scale to compute relevancy in search engines (16).

We present a medium-scale tokenized and sentence-split document corpus derived from PubMed and PMC that is also filtered for molecular studies using high-level Medical Subject Heading (MeSH) terms. We provide word embedding models using GloVe, FastText and ELMo methods and then investigated performance of simple classifier architectures for two evidence-driven biocuration tasks: (i) identifying the detection methods used in individual molecular interaction experiments and (ii) document-level classification (‘triage’) for experimental studies containing legitimate work describing new interaction data. We sought to investigate a possible role for evidence-based language from articles’ results sections pertaining to figures for these two text classification tasks. This work forms an early component of a strategic investigation into ‘evidence extraction’: focused machine reading methods targeting scientific evidence from figures and associated text.

## Materials and methods

### The molecular open access PubMed document corpus

Every paper indexed into PubMed is tagged with MeSH codes (https://www.nlm.nih.gov/mesh/) that provide basic conceptual tagging by a human curator. In order to limit our domain of discourse to molecular bioscience, we ran searches over PubMed and PMC based on a set of high-level terms within the MeSH controlled vocabulary hierarchy that pertained to molecular work. These are listed here:

(i) Cells [A11]

(ii) Multiprotein Complexes [D05.500]

(iii) Protein Aggregates [D05.875]

(iv) Hormones [D06]

(v) Enzymes and Coenzymes [D08]

(vi) Carbohydrates [D08]

(vii) Lipids [D10]

(viii) Amino Acids, Peptides and Proteins [D12]

(ix) Nucleic Acids, Nucleotides and Nucleosides [D13]

(x) Biological Factors [D23]

(xi) Pharmaceutical Preparations [D26]

(xii) Metabolism [G03]

(xiii) Genetic Phenomena [G06].

**Figure 1:**
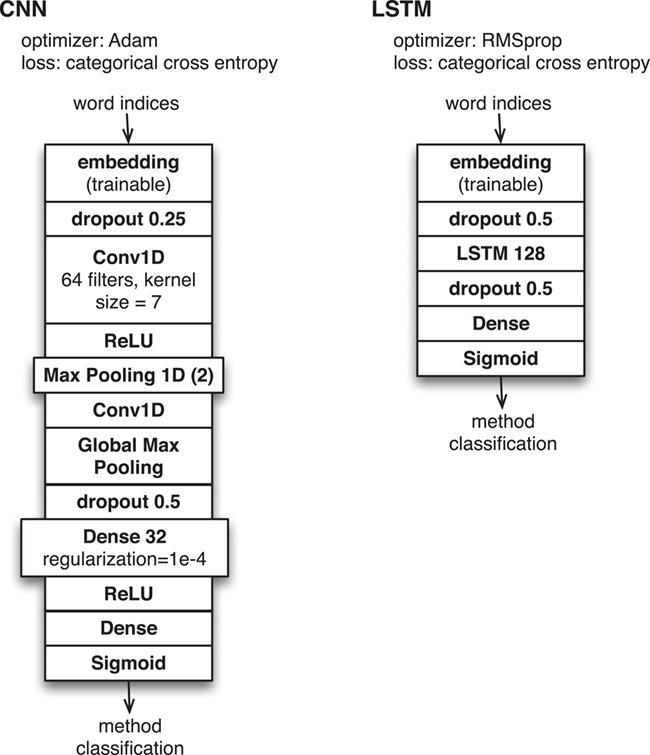
Neural network configurations for method classification based on INTACT evidence fragment corpus.

Executing this query on PubMed returns 11,447,521 abstracts. Similarly, we retrieved 1,720,266 full-text document identifiers from PMC, 509,722 of which are open access. We downloaded, parsed and concatenated 403,825 PMC open access PMC documents, combined with all relevant PubMed titles and abstracts. We concatenated the text from the two queries into a single 30 GB file where each line is a single sentence, and the text is fully tokenized (17). These preprocessing steps were implemented using the ‘UIMA-Bioc’ software library that uses the ClearTk sentence splitter and tokenizer (with corrections for headings and titles that do not end in periods, see https://github.com/SciKnowEngine/UimaBioC).

### Word embeddings

We computed word embeddings using provided tools for GloVe, FastText and ELMo based on the corpus described in Burns *et al*. (2018) (17) as well as using word embedding data trained on non-biomedical text (GloVe and FastText). We trained models with 50, 100, 300, and 1024 dimensions for GloVe as well as 100 dimensions FastText based on the molecular open access PubMed document corpus in order to explore performance across the models on the classification tasks described. Training the ELMo model is computationally expensive, and we created only one available embedding dataset with 3 × 1024 dimensions based on two epochs of the training process. A key contribution is a complete, well-documented file distribution for use by the community for biocuration application development (17).

### INTACT detection method prediction

We followed the approach described in Burns *et al*. (2018) (18) where curated PSI-MI2.5 data from the INTACT data records are linked to text from captions and the main text body from the paper via the designated subfigure of the data record (i.e. ‘Figure 1C’, etc.). This application of distant supervision (19) allows us to attempt to train a machine learning model that can predict the type of methods being used based on text associated with a given subfigure. As with Burns *et al*. (2018) (18), we used PSI-MI2.5 codes for interaction or participant detection methods as classification targets, reducing the number of available targets from 44 finely delineated participant detection codes to 8 grouped codes and from 84 interaction detection method codes to 17 grouped codes. We also investigated the classifier’s performance at a simple yes/no classification task for specific common experimental methods: (i) use of western blot methods for participant detection and (ii) use of coprecipitation methods for interaction detection. Training data for these tasks are available from the Zenodo repository (20).

We developed deep neural network classification software that could use any of the available word embedding models as input for one of several available neural network configurations based on (i) long short-term memory (LSTM) and (ii) convolutional neural network (CNN). These correspond to the same level of generic, unsophisticated models that are provided as examples from standard documentation for the Keras deep learning library (see https://keras.io) and are shown in detail in Figure 1. For these machine learning experiments, we ran multiple executions of all classifiers with randomized initialization settings to control for variability and local minima in the training process.

### Document triage for molecular interaction with ‘Darkspace’ data

The ‘Darkspace Project’ is an initiative from the INTACT biocuration team to develop technology capable of finding previously unseen papers from the scientific literature containing molecular interaction data (https://github.com/pporrasebi/darkspaceproject). We are grateful to Dr Pablo Porras for providing a list of 537 positive and 451 negative training examples of open access papers that either contain or do not contain scientific information pertaining to molecular interactions. We queried PubMed to obtain title, abstract and MeSH data for each paper. We downloaded ^*^.nxml-formatted files from PMC and invoked the UIMA-Bioc preprocessing pipeline (as described previously) to extract figure captions and ‘evidence fragment’ text pertaining to each subfigure mentioned in the textual narrative (21). Given these data, we performed document-level text classification as a ‘triage’ task (i.e. to assist biocurators to identify papers of interest). In particular, we hypothesize that evidence-based text would be more informative in that classification than text generally derived from the paper as a whole.

As a baseline, we used a simple CNN to classify document-level text such as the title, abstract and MeSH keywords. We wanted to judiciously use the text from within the full text of the paper to help the system make its triage decision, but found that the memory requirements of the system prevented us from naively classifying the entire full text. We therefore investigated methods of breaking the full text into paragraphs and then used neural network attention methods to learn an aggregated signal that formed the basis of the final document-level classification. We investigated four cases (i) using each paragraph in the full-text document, (ii) using each ‘evidence fragment’ (i.e. the text surrounding a figure reference that directly describes the figure) (21), (iii) using each figure caption and (iv) using evidence fragments and captions together. We broke the document-level text into collections of paragraphs so that the network classifier could make inferences aggregated over classification decisions made for each individual paragraph. More technically, the network architecture is based on a CNN-Bidirectional LSTM model for each paragraph, augmented with attention to convert the array of paragraph-level vectors into a single vector for whole document and then completed with a fully connected layer to convert the representation to a triage decision. As with our method classification problem, we focus on the use of simple, easy-to-implement machine learning models that could be easily replicated by researchers without deep knowledge of NLP. We tested a variety of machine learning methods over available word embedding models to determine classification performance.

### Source code distribution

In order to render this work as accessible and useful as possible, all code and data are available as open source and are described on aggregate as a Research Object (22) with the following URI: http://purl.org/ske/ro/biocuration2019. This is a relatively simple aggregation of persistent URIs for all source code and data used to generate this paper. In particular, we highlight the code distribution associated with the GitHub repository associated with this project: https://github.com/SciKnowEngine/evidX. We use Docker to build the core functionality of our system as components that execute on well-defined virtualized containers, ensuring that the complexities of managing dependencies and other issues are not a barrier to reusing our code.

## Results

### Exploring molecular interaction method prediction

**Figure 2:**
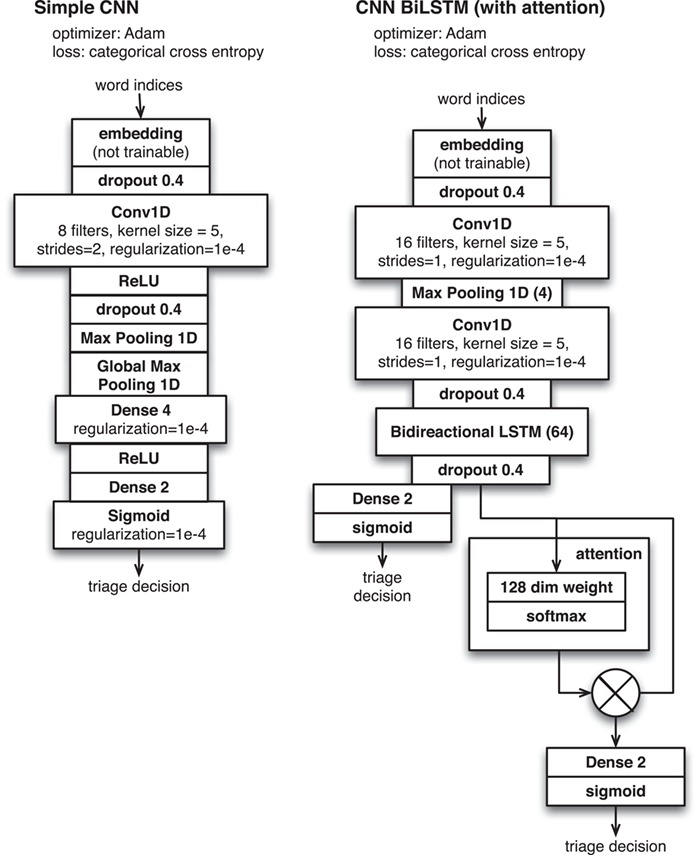
Neural network configurations for document triage applied to ‘Darkspace’ data.

Here we describe the task of predicting the PSI-MI2.5 codes for ‘participant detection method’ and ‘interaction detection method’ for specified subfigures in the INTACT database (based only on open access data). We sought to characterize the performance of several different standard neural network approaches in order to support the use of these tools across various similar focused text classification tasks of relevance to biocuration. This directly builds on previous work performing the same classification task with non-deep learning methods (21) or with FastText classifiers as part of a broader workflow (18). We here examine classification performance for all available word embedding models under a variety of parameterizations (see Figure 2).

Based on these experiments, we make a number of claims. For this task (method classification), text of figure captions performed better than the text of evidence fragments (compare ‘Caption + LSTM + BioGlove 300’ to ‘Evidence Frag. + LSTM + BioGlove 300’ in Figure 2). This is consistent with earlier findings (23). Embeddings trained on bio-domain generally perform better than general-purpose embeddings (compare ‘Caption + LSTM + BioGlove 300’ to ‘Caption + LSTM + Glove 300’ in Figure 2). Among the BioGloVe embeddings the 300-dimensional model had the best performance (see the ‘Caption + LSTM + BioGlove’ series across 50, 100, 300, and 1024 dimensions in Figure 2). The LSTM classifier generally performs marginally better than the CNN (see the ‘Caption + LSTM + BioGlove 300’ vs. E‘Caption + CNN + BioGlove 300’ series in Figure 2). Finally, in these experiments we found that the BioGlove embeddings performed marginally better than the FastText embeddings (see the ‘Caption + LSTM + BioGlove 300’ vs. ‘Caption + LSTM+ Bio Fasttext 100’ series in Figure 2).

### Document triage for molecular interaction with ‘Darkspace’ data

We examined the application of relatively standard deep learning tools based on a variety of word embeddings trained from the molecular open access corpus. We found that, of the embedding configurations we used, we obtained best performance from a 100-dimensional FastText model, which narrowly surpassed performance of a 50-dimensional GloVe model. Still, higher-dimensional representations of GloVe models performed less well. As described in the Methods section, we applied an attention mechanism over designated passages from the paper to make optimal use of the full text to classify the whole document. In this way, we trained a classifier over (i) all paragraphs in the document, (ii) all caption text from the document, (iii) all extracted evidence fragments from the paper and (iv) the combined captions and evidence fragments.

As shown in Table 1, this aggregated method of processing full text outperforms classifiers trained on the abstract or the title and abstract combined. There is also some indication that leveraging the text that directly describes evidence (in figure captions and text surrounding figure references) consistently outperforms analyses with all text in the document across representations. We found better performance overall with 100-dimensional FastText embeddings with the 50-dimensional GloVe embeddings coming in a close second. As was the case with method classification, the bio-derived word embeddings marginally outperform larger word embeddings trained on general text.

**Table 1:** Triage Accuracy by text source, network model, and embedding type

**Text source**	**Model configuration**	**Triage accuracy**	
		**FastText-100**	**GloVe-50**
Abstract	CNNBiLSTM	0.71 ± 0.02	0.76 ± 0.02
All paragraphs	CNNBiLSTM + attention	0.81 ± 0.01	0.76 ± 0.01
Captions	CNNBiLSTM + attention	0.82 ± 0.01	0.79 ± 0.01
Captions + Evid. Frg.	CNNBiLSTM + attention	0.82 ± 0.01	0.77 ± 0.01
Evid. Frg.	CNNBiLSTM + attention	0.81 ± 0.01	0.77 ± 0.01
MeSH	Simple CNN	0.61 ± 0.03	0.70 ± 0.02
Title	Simple CNN	0.60 ± 0.02	0.69 ± 0.02
Title + Abstract	CNNBiLSTM	0.72 ± 0.01	0.71 ± 0.01

**Figure 3:**
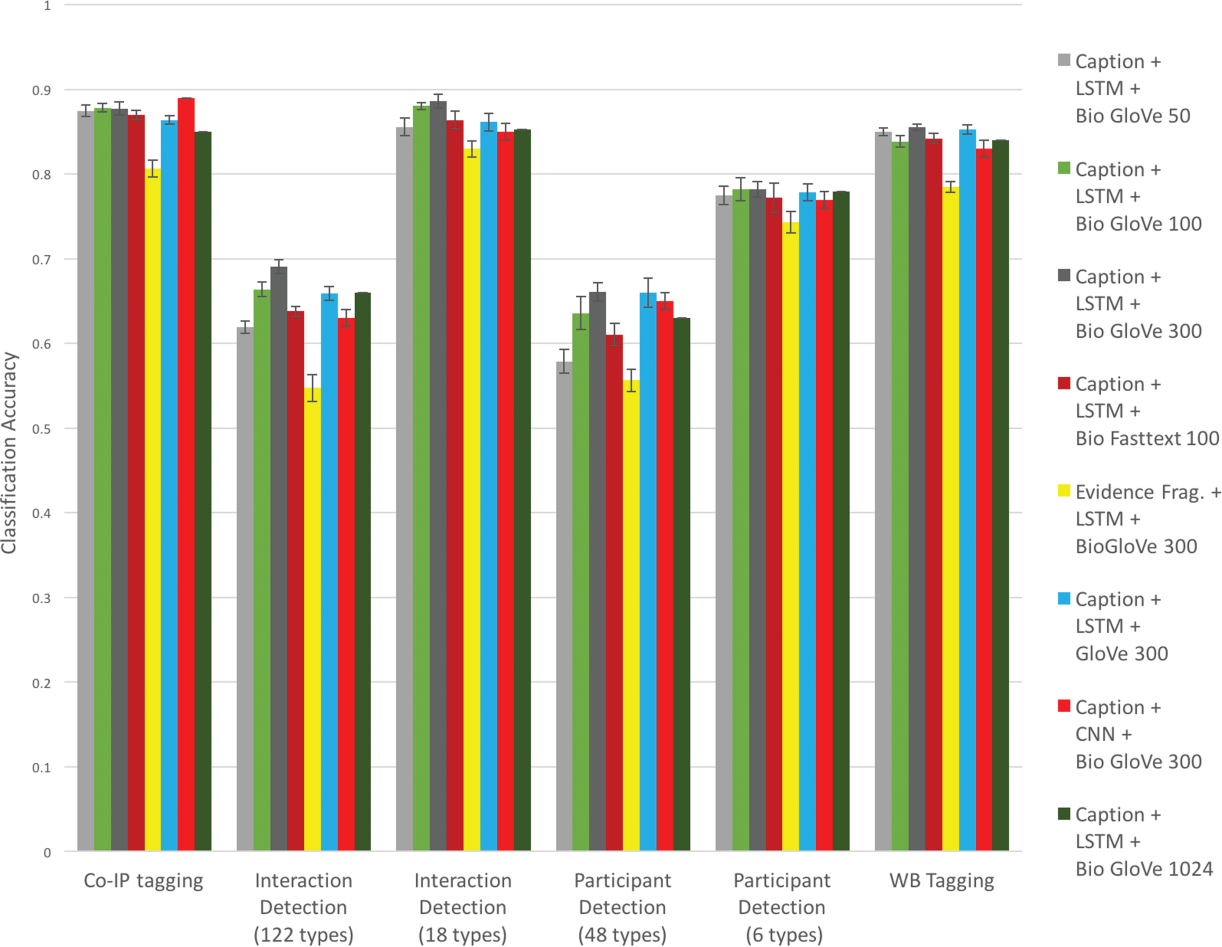
Classification accuracy for experimental methods based on text, network model, and embedding.

### Experiences with deep contextualized word representations

ELMo was shown to be more efficient than other word embeddings in various NLP tasks such as named entity identification, semantic role labeling (9), as it uses three layers of recurrent neural networks to efficiently model the context information and thus efficiently disambiguate words in the contexts. However, in our experiments, ELMo struggles to perform even on par with GloVe and FastText embeddings. One reason lies in the fact that ELMo is a computationally expensive model. We trained it for 4 months with 2 graphics processing units (GPUs) but only finished 2 epochs of training, while 1024-dimensional GloVe embedding training converged at 20 epochs within a day. Similarly, it is hard to further fine-tune the ELMo embeddings to tailor the downstream tasks as we do for other embeddings due to its computational complexity. Efficient algorithms are desirable for training richer contextualized word embeddings for biomedical tasks. We found that the training process was extremely resource hungry, even with access to a large (by academic standards) local GPU-enabled cluster. We were able to train a partial model but were not able to generate competitive scores in either of the tasks we described here. Clearly, this aspect of the use of deep learning presents a possible difficulty for academic biocuration work.

## Discussion

In this paper, we have applied deep learning methods to biocuration-driven NLP tasks. Part of the motivation for this is that deep learning techniques can be used to train classifiers with state-of-the-art performance with relatively little training data (given the existence of an effective word vector representation). We sought to investigate the use of these methods for two, well-defined tasks in a specialized subdomain: (i) identifying the type of an experiment based on text from figure captions or relevant sections of the results sections and (ii) document triage as described by the ‘Darkspace Project’. A key aspect of both of these experiments is our focus on the role of evidence as a signal to be detected using NLP, either directly as with method type classification or indirectly with the triage task.

The main conclusion we have regarding the technical implementation of these systems is that the choice of the model structure and the embedding depends on the length of the text being classified. Paragraph-level texts (300 words or less) are desirable. We found that the model would predict perfectly on training data but could not perform equally well on previously unseen testing data. This was mitigated by reducing the number of trainable parameters, such as making the embedding layer not trainable or reducing the neural network size. Adding dropout layer with appropriate dropout rate (from 0.25 to 0.5 in our case) and regularization (1e-4 in our case) also improved performance. Crucially, if the length of the text being classified size is greater than 300 words, the use of attention mechanisms can produce high performance at document-level classification tasks. Our work mainly focused on comparing the effect of using different embedding layers on top of several naive neural networks architectures such as CNN and LSTM. These suboptimal architectures may be one of the reason that results in poor performance. For the future work, more investigation can be made on exploring proper neural network architectures that better capture input features and improve performance. For example, the attention mechanism (24; see discussion) can be applied on LSTMs to better summarize word representations to make more accurate predictions.

As shown in Figure 3, we were able to obtain excellent classification performance to identify interaction detection methods based on caption text. At best, our system achieved accuracy of 0.89 using LSTM and BioGlove 300-dimensional input vectors on caption text when the number of target categories was reduced from 122 to 18. Captions are easy to locate within full-text documents and we anticipate that this approach could form the basis of functional tools. Within the evidence extraction project at ISI (https://github.com/SciKnowEngine/evidX/), we intend to use this approach to help us automatically identify experimental design templates for published molecular interaction experiments based on our ‘Knowledge Engineering from Experimental Design’ methodology (25).

Triage is a task that has justifiably attracted much attention from NLP researchers over the years including the shared TREC competitions in 2004 (26) and 2005 (27). In 2004, the top-scoring system had an F-Score of 0.6512, and no system was able to substantially improve results over simply using the MeSH term ‘Mice’ (with some improvement the following year). Performance in triage tasks is typically strongly dependent on the inclusion criteria being specified to denote a document as being of interest to the target system. For example, attempting to design a triage system to detect papers based on criteria that requires expert human interpretation to determine is likely to be very difficult to automate (as was the case with the TREC 2004 challenge where papers were judged for having a mouse-based phenotype). Our work investigates the strategy of leveraging text describing evidence as a powerful signal for document classification with an upper bound of 0.82 accuracy based on caption text. This is encouraging. We expect to be able to improve this by (i) providing more training data, (ii) improving the delineation of experimental fragments that currently is based on a simple heuristic algorithm and (iii) further investigating the role of attention in identifying which intermediate, learned features to base the final triage decision on. Attention is a powerful construct in deep learning, where systems may learn which parts of a long signal are most influential in making downstream decisions (24). In the triage application, many parts of a full-text paper may have some bearing on a curator’s decision to include it in his workflow. The ability to automatically identify which parts of the paper are most relevant would suggest that studying attention as part of document triage could have great value in developing effective tools for biocurators.

It is worth noting that the curation rules provided by the IMEX consortium (http://www.imexconsortium.org/) do not provide explicit inclusion/exclusion criteria for molecular interaction papers except to say that ‘a complex or set of interacting molecules has at least been partially purified’. There are many inherent aspects of experiments described in the guidelines that could only be internalized and used by an expert human biocurator. Clearly, the necessary semantic information to make such a decision is deeply embedded in domain-specific knowledge of the domain.

The difficulties of developing effective ELMo models for either task present a serious challenge to this work but we remain quite optimistic about the potential impact of developing this resource for the community. An important part of our work is centered on developing reusable resources for biomedical developers that are tailored to biomedical data needs. Our efforts with the molecular open access PMC corpus (17) is based on all available open access documents indexed in PMC with molecular-based MeSH terms. As part of this work, we also make available GloVe and FastText embeddings for use in the community. The potential power of these richly contextualized models could provide still further improved capabilities for biocuration tasks but the sheer computational expense of constructing ELMo models is proving to be a serious obstacle.

More broadly, these modern deep learning models have very broad applicability to NLP in the life sciences beyond the scope of the biocuration task. Our work serves as a demonstration of the potential utility of deep learning models across a range of more general applications (such as document classification, automated or semi-automated IE). The rapid progress made underlying the development of high-quality representation models such as BERT is encouraging and should be pursued.

## Notes

**Database URL:** http://purl.org/ske/ro/biocuration2019
